# PHACE syndrome: looking backward and forward

**DOI:** 10.1186/s13023-025-03899-7

**Published:** 2025-07-07

**Authors:** Kaizhi Zhang, Shanshan Xiang, Jiangyuan Zhou, Tong Qiu, Yuru Lan, Yi Ji

**Affiliations:** Division of Oncology, Department of Pediatric Surgery, West China Hospital of Sichuan University, No. 37 Guo Xue Alley, Chengdu, 610041 China

**Keywords:** PHACE syndrome, Infantile hemangioma, Pathogenesis, Risk factors, Diagnosis, Treatment

## Abstract

PHACE syndrome rarely occurs in patients with infantile hemangioma (IH) but is common in patients with segmental IH involving the head and face. PHACE syndrome involves at least one system abnormality, including arterial abnormalities, structural brain abnormalities, cardiovascular abnormalities, eye abnormalities, and ventral or midline abnormalities. The pathogenesis of PHACE syndrome remains unclear, and it affects various systems in diverse ways. Oral propranolol has good effects on patients with PHACE syndrome. However, there are great challenges in the management of patients with PHACE syndrome in the later stage, including headaches, stroke, neurodevelopment impairment, psychosocial impacts, and poor quality of life. Therefore, this review summarizes the epidemiology, risk factors, pathogenesis, clinical manifestations, diagnosis, treatment, and complications of PHACE syndrome. The latest research on and therapeutic prospects for PHACE syndrome are also discussed.

## Introduction

**Fig. 1:**
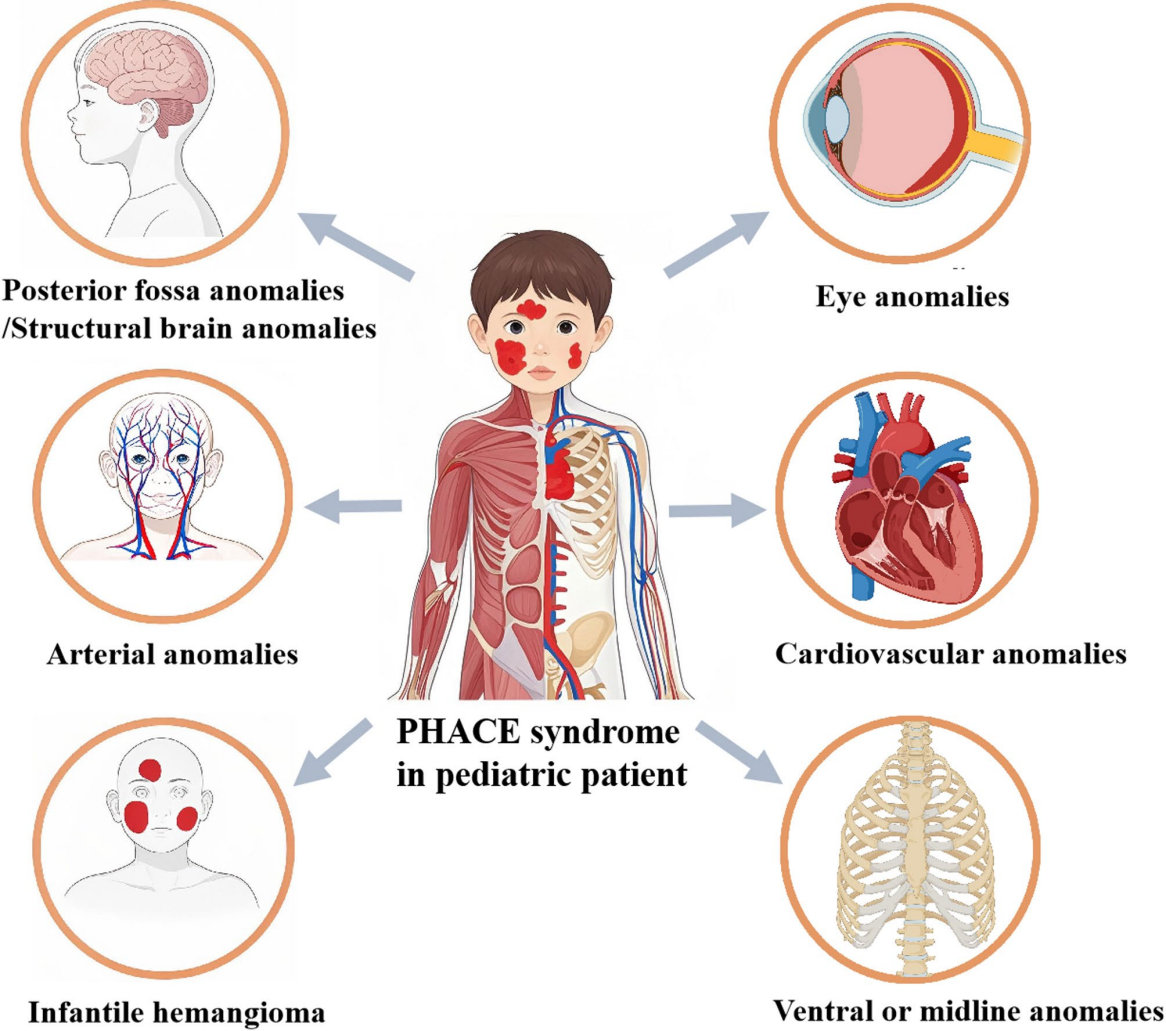
Diagram of the organ systems involved in PHACE syndrome

**Fig. 2:**
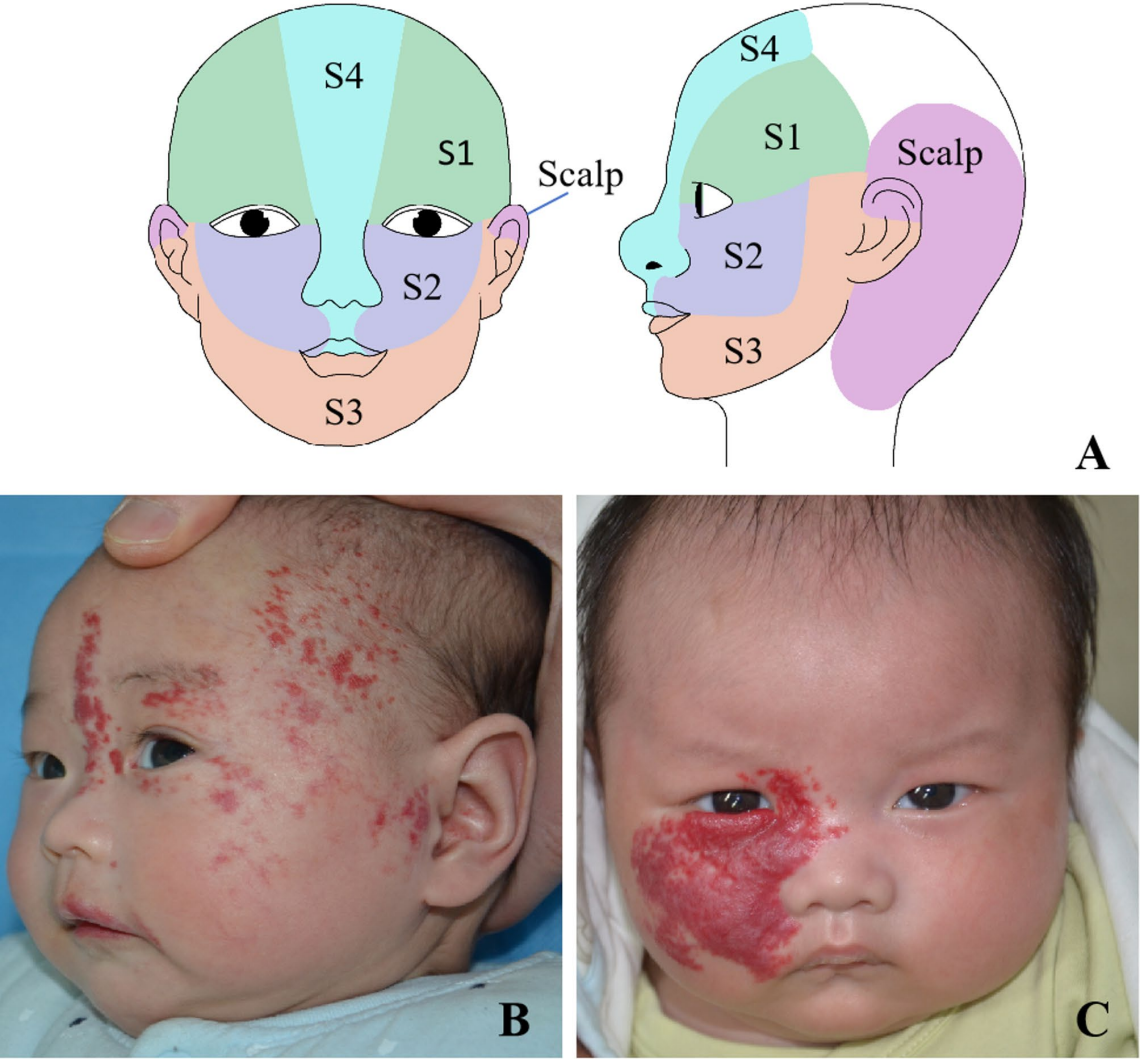
Pictures of patients with PHACE syndrome. **A**: Facial segment distribution adapted from Endicott et al., 2021 [8]. **B**: A 3-month-old female with PHACE syndrome with large left facial IH involving S1, S2, S3, and S4. **C**: A 2-month-old male with PHACE syndrome with large right facial IH involving S1, S2, and S4

PHACE syndrome is a neurocutaneous syndrome involving multiple system abnormalities, including posterior fossa anomalies, hemangioma, arterial lesions, cardiac abnormalities/coarctation of the aorta, and eye anomalies [1]. In 1996, Frieden et al. [2] combined the initials of each lesion system and used ‘‘PHACE’’ to describe the disease for the first time (Fig. 1). Further investigations have shown that a subset of patients with PHACE syndrome exhibit abnormalities in the ventral or midline region, endocrine disorders, and dental issues [3–5]. The main feature of PHACE syndrome is segmental infantile hemangioma (IH) in the head and face. Clinically, the overall incidence of PHACE syndrome is low for patients with IH but high for segmental IH in the head and face [6]. In 2006, Haggstrom et al. [7] divided the anatomical position of the head and face and split segmental IH into 4 regions: S1 (frontotemporal), S2 (maxillary), S3 (mandibular) and S4 (frontonasal). In 2021, Endicott et al. [8] revised the boundary between S2 and S3, and added a novel C-shaped pattern of scalp (Fig. 2). Recent research on PHACE syndrome has increased our understanding of the disease, including its incidence, pathogenesis, risk factors, clinical manifestations, diagnosis, treatment, complications, and long-term follow-up. This review mainly describes the current understanding of PHACE syndrome and therapeutic prospects, with the aim of increasing clinicians understanding of PHACE syndrome.

### Epidemiology

PHACE syndrome is rare, accounting for approximately 2.3% of IH [9]. Hemangiomas in PHACE syndrome usually present as large red plaques involving multiple segments, followed in frequency by plaques on the right side (29%) and on the bilateral sides (27%) [10]. Studies reported that 20–31% of patients with segmental IH in the head and face have PHACE syndrome, which was more prevalent in females, with a male-to-female ratio of up to 1:9 [6, 9, 11]. In recent years, the reported incidence of PHACE syndrome has increased due to the revision of diagnostic criteria and enhanced understanding among medical professionals. Cotton et al. [12] and Forde et al. [13] reported that 44.5% (108/238) and 58% (29/50) of cases of segmental IH in the head and face were accompanied by PHACE syndrome, respectively. The incidence of PHACE syndrome is high in Europe and North America, with most patients being white or non-Hispanic, and most patients have full-term births and normal birth weights [11, 12, 14]. However, little is known about the actual incidence of PHACE syndrome in Asia and Africa. Studies indicated that the incidence of IH was greater among white populations than among Asian and Black populations [15–17]. Therefore, the prevalence of PHACE syndrome in Asia and Africa may be lower than that reported in Europe and North America.

### Risk factors

The risk factors for IH may be genetic, maternal, or fetal, but the risk factors for PHACE syndrome are unclear [18, 19]. Some studies reported that there were no significant differences in maternal factors between patients with and without PHACE syndrome [9, 11, 20]. Wan et al. [14] reported that preeclampsia and placenta previa may represent potential risk factors for PHACE syndrome, but it was unclear whether they have a direct effect on abnormalities in various systems. Further research is necessary to elucidate this relationship.

The larger the area of segmental IH in the head and face is, the greater the possibility of PHACE syndrome. The involvement of S1, S4, or ≥ 2 segments is also closely related to the occurrence of PHACE syndrome [9, 11, 21]. However, a recent multicenter retrospective study revealed that an IH surface area ≥ 25 cm^2^ and involving ≥ 3 segments were high-risk factors for PHACE syndrome. The involvement of the neck, parotid gland, or S2 segment was a low-risk factor, and race and ethnicity might be related to PHACE syndrome [12]. These findings are crucial for parents and doctors in assessing PHACE syndrome comprehensively. However, the risk factors for PHACE syndrome remain incompletely understood. Further large-scale, prospective, and multicenter studies are needed.

### Pathogenesis

**Fig. 3:**
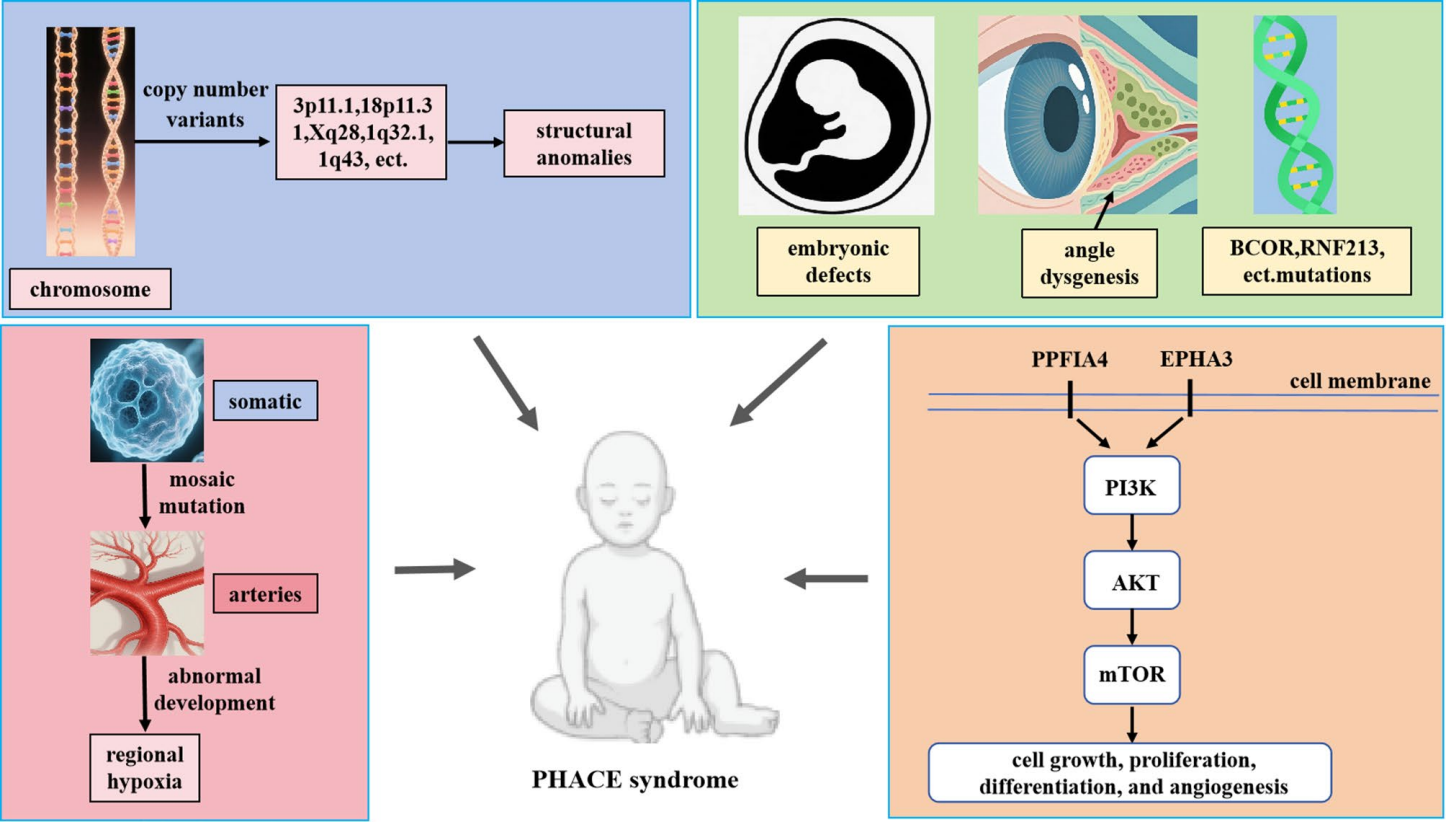
Diagram of the possible pathogenesis of patients with PHACE syndrome. Copy number variants, somatic mosaic mutations, and gene dysfunction are the primary pathogeneses. Other factors may include embryonic development defects, abnormal vascular development, and regional hypoxia

Overall, the etiology of PHACE syndrome remains unclear and may be associated with a multifactorial interplay of genetic and developmental factors (Fig. 3). Hypoxia is one of the main causes of IH [22, 23]. Some scholars have suggested that PHACE syndrome may also be related to regional hypoxia secondary to abnormal vascular development [24, 25]. Moreover, some studies have hypothesized that PHACE syndrome may be related to X chromosome inactivation and X chromosome gene mutation [26, 27], but a study by Sullivan et al. [28] did not support that hypothesis.

The abnormalities associated with PHACE syndrome may be related to embryonic development defects at 6 to 8 weeks of pregnancy [10, 29, 30]. Some studies have suggested that somatic mosaic mutations in neural crest-derived cells lead to changes in blood vessels [7, 31] and that these cells then migrate along certain metamerism segments under the control of the HOX gene [32]. Therefore, the anomalies along the same side of these fragments can be explained by the same mutation [33]. The BCOR gene is important for embryogenesis and circulatory system development. The BCOR gene may be a key regulator of development and tumorigenesis, especially in IH, and it may be related to PHACE syndrome [34]. One study performed whole-genome sequencing of samples from patients with PHACE syndrome and their parents was performed, and rare mutations in stromal cell signaling genes were found in some patients [35]. Because the frequency of mutant alleles with mosaic mutations is very low, no single pathogenic gene for PHACE syndrome has been identified to date [3]. Further analysis using next-generation sequencing may reveal pathogenic mutations associated with PHACE syndrome.

Copy number variants (CNVs) may play important roles in PHACE syndrome. In 2013, Siegel et al. [36] used an Affymetrix gene chip 6.0 single-nucleotide polymorphism array to study CNVs in 98 patients with PHACE syndrome. This study revealed that 10 rare CNV regions, including 1q32.1, 1q43, 3q26-3q26.33, 3p11.1, 7q33, 10q24.32, 12q24.13, 17q11.2, 18p11.31, and Xq28, presented many rare copy increases and copy losses. However, these alterations only appeared in a single individual, not among multiple individuals. CNV regions were identified at 3p11.1 in a patient presenting with sternal clefts and supraumbilical raphes, as well as at 18p11.31 in another patient exhibiting posterior fossa anomalies and arterial anomalies. Certain CNV regions encompass genes involved in the hypoxia inducible factor-1ɑ (HIF-1ɑ), vascular endothelial growth factor (VEGF), and mammalian target of rapamycin (mTOR) signaling pathways (such as VTN, PPFIA4, EPHA3, and EMILIN2), suggesting that these genes may play a role in the pathogenesis of PHACE syndrome [36]. The contribution of these CNVs to the pathogenesis of PHACE syndrome warrants further investigation.

Other possible pathogeneses include angle dysgenesis, abnormal mesodermal vascular endothelial cell cloning, placental chorionic mesenchymal stem cell implantation into neural crest cells during early embryonic development, TMEM260 compound heterozygous variants, and RNF213 variants [4, 5, 11, 37–40]. In the future, larger-scale research will help clarify the pathogenesis of PHACE syndrome.

### Manifestations of PHACE syndrome

#### Arterial anomalies

**Fig. 4:**
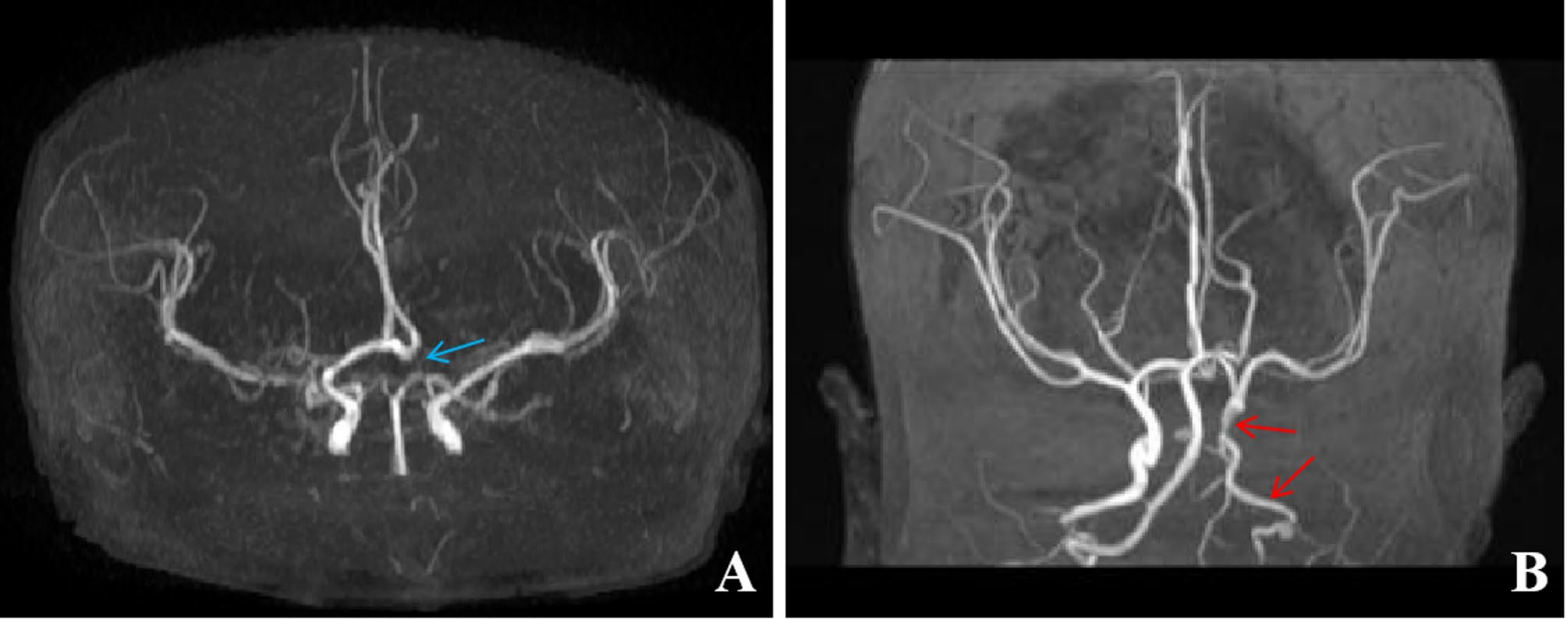
Representative images of patients with PHACE syndrome. **A**: MRA image of a 4-month-old female with PHACE syndrome showing the absence of the left anterior cerebral artery (blue arrow). **B**: MRA image of a 24-day-old female with PHACE syndrome showing intracranial stenosis of the left internal carotid artery and vertebral artery (red arrows)

Arterial abnormalities are the most common extracutaneous lesions of PHACE syndrome, occurring in 63.6–91.3% of PHACE syndrome [6, 9, 21, 41, 42]. PHACE syndrome with S1 segment involvement of the face are at increased risk of cerebral artery abnormalities [11, 21]. Most vascular diseases involve large and medium blood vessels in the neck and brain (such as the internal carotid artery, anterior cerebral artery, vertebral artery, middle cerebral artery, and posterior communicating artery), which can manifest as arterial dysplasia, arterial stenosis or occlusion, arterial absence or hypoplasia, arterial distortion, or cerebral aneurysm [43–45] (Fig. 4). The abnormal part of the cerebral artery is usually located on the same side as the IH in the face, and anatomical variation in the circle of Willis can be observed in 18.1% of PHACE syndrome [21]. A multicenter long-term follow-up study revealed that among 68 patients with PHACE syndrome with imaging data, 29.4% had vascular progression, and 8.8% had moyamoya disease or progressive arterial stenosis or occlusion [41].

Clinicians need to identify arterial abnormalities during the process of diagnosis and treatment. Arterial abnormalities pose a great threat to patients, especially in terms of the risk of progressive arterial disease [44]. Some patients with PHACE syndrome have progressive vascular disease. Despite the slow progression of vascular disease, regular monitoring through cerebral magnetic resonance angiography (MRA) is crucial for patients to select appropriate medications or surgical interventions. MRA should be performed within one month of birth to assess baseline arterial abnormalities, with subsequent imaging surveillance extended to 2 to 3 years or longer in suitable cases [45–47]. Abnormal cerebral arteries and carotid arteries increase the risk of arterial ischemic stroke, with such patients being divided into three risk groups (low-, medium-, and high-risk groups) according to severity, as determined by MRA [44, 48, 49]. The establishment and management of risk groups should involve multidisciplinary experts, including neurologists, neuroradiologists, vascular specialists, and stroke experts.

#### Structural brain anomalies

Structural brain anomalies are common in PHACE syndrome. In recent years, abnormal brain structure has been reported in 36.4–81% of patients with PHACE syndrome [12, 21, 41, 44]. Structural brain abnormalities include posterior fossa abnormalities, Dandy‒Walker complex, midbrain or hindbrain hypoplasia, midline abnormalities, and cortical dysplasia. The common structural brain abnormalities include posterior fossa abnormalities and midbrain or hindbrain hypoplasia, followed by Dandy‒Walker complex abnormalities, midline abnormalities, and cortical dysplasia [12, 41, 45]. The Dandy‒Walker complex is a typical abnormal brain structure that is characterized by enlargement of the posterior fossa, elevation of the tentorium cerebelli, and cystic dilatation of the fourth ventricle. However, some studies suggest that unilateral cerebellar hypoplasia is a more characteristic brain abnormality [50, 51]. Facial IH and brain structural diseases are usually located on the same side, and S1 segment involvement is closely related to abnormal brain structure [11, 21, 27]. Brain structural diseases and cerebrovascular diseases usually coexist in patients with PHACE syndrome, but nonvascular brain diseases rarely coexist with PHACE syndrome (posterior fossa, midbrain, and hindbrain), with only 5 cases reported at present [33, 52, 53].

Some studies have emphasized that prenatal examinations (such as ultrasound and magnetic resonance imaging [MRI]) can be used to identify fetuses with PHACE syndrome. However, the number of patients in those studies was small, and the specific predictive value was uncertain [54–56]. We hypothesize that the possibility of prenatal diagnosis depends more on the extent of multisystem organ involvement and whether a specific individual has findings that can be detected with prenatal imaging.

#### Cardiovascular anomalies

Cardiovascular abnormalities are among the important criteria for the diagnosis and are relatively common in PHACE syndrome, affecting approximately 27–67% of cases [11, 21, 42, 48, 57]. The common cardiovascular abnormalities are aberrant subclavian artery anomalies with or without vascular rings and coarctation of the aorta, followed by aortic arch anomalies and ventricular septal defects. Cardiac aneurysms and systemic venous anomalies are rare in PHACE syndrome [41, 48, 58–60]. Patent foramen ovale and patent ductus arteriosus are common in patients with PHACE syndrome [35, 59], and pulmonary artery stenosis, atrial septal defects, ectopia cordis, and Holmes’ heart are also found in some patients [61, 62]. However, these abnormalities are not criteria for the diagnosis of PHACE syndrome.

Segmental IH involving the S1 and S3 segments may be closely related to cardiovascular abnormalities, and patients with S3 segment involvement have a greater risk of cardiovascular malformations [11, 63–65]. One prospective study reported that 61% of patients with PHACE syndrome had both cardiovascular and central nervous system abnormalities and a high prevalence of coarctation of the aorta [11]. In a European multicenter cohort study of PHACE syndrome by Disse et al. [66], cardiovascular abnormalities accounted for 50% of all abnormalities, among which coarctation of the aorta was common, and most patients required treatment, including intervention or surgery. Coarctation of the aorta due to PHACE syndrome is not the same as coarctation due to other causes, and Bayer et al. [59] identified different patterns in the location and histopathological features of coarctation of the aorta in patients with PHACE syndrome, revealing a unique pathogenesis. The incidence of cardiovascular anomalies is second only to that of cerebrovascular anomalies [33, 52, 53]. Therefore, in patients with suspected PHACE syndrome, not only brain MRI/MRA examinations but also echocardiography should be performed to identify abnormalities in the cardiovascular system in a timely manner, which is beneficial for subsequent diagnosis and treatment.

#### Eye anomalies

The true incidence of ocular abnormalities meeting diagnostic criteria in patients with PHACE syndrome is unknown. Ocular abnormalities have been reported in approximately 6–26% of patients with PHACE syndrome [6, 41, 57]. The incidence of ocular abnormalities reported in different studies varies significantly, possibly due to the lack of ophthalmic referrals and direct ocular examinations, especially in the absence of periocular hemangioma or macroscopic abnormalities. Patients with S1 and/or S4 segment involvement may be associated with ocular abnormalities [9, 35, 50]. Ocular abnormalities meeting diagnostic criteria in patients with PHACE syndrome are classified into two conditions: those with periocular IH involvement and those without periocular IH involvement. In a follow-up study of 30 patients with PHACE syndrome over 15 years, Samuelov et al. [67] reported that most ocular complications were secondary to the periocular IH location itself and rarely caused by PHACE syndrome. Another study revealed that 67% (29/43) of patients with PHACE syndrome had periocular IH and that five of these patients met the PHACE diagnostic criteria for ocular abnormalities, including abnormalities of the optic nerve, retinal vessels, and lens [57]. PHACE syndrome patients without periocular IH involvement may also present with ocular abnormalities [68], and abnormalities in the posterior segment of the eye are often accompanied by intracranial vascular abnormalities [50]. Therefore, it is recommended that patients with segmental IH in the head and face undergo ophthalmologic screening regardless of the presence or absence of periocular IH.

Other ocular symptoms, including amblyopia, ptosis, decreased vision, increased intraocular pressure, and limited eye opening, are also relatively common in PHACE syndrome patients [66]. One study reported that among 21 patients with PHACE syndrome with periocular IH, 43% presented with ptosis or ametropia at the time of diagnosis, and one patient presented with Horner syndrome [50]. However, no ocular abnormalities were observed in a study by Lamotte et al. [65]. It is hypothesized that early propranolol treatment can reduce the volume of periocular IH and reduce the occurrence of ocular complications.

#### Ventral or midline anomalies

The incidence of ventral or midline anomalies in patients with PHACE syndrome ranges from 6.7–26% [41, 57]. Ventral or midline abnormalities include mainly sternal defects, sternal pits, sternal clefts, and supraumbilical raphes, as well as rare cases of ectopic thyroid hypopituitarism, midline sternal papules, and hamartomas [44, 69]. Patients with PHACE syndrome involving the S3 segment are more likely to have ventral or midline abnormalities [9, 27].

Feigenbaum et al. [70] observed that patients with segmental IH can see the midline turning white without obvious ventral or midline abnormalities, which was a risk factor for PHACE syndrome. Therefore, midline whitening may be a manifestation of ventral defects. Some scholars believe that abnormalities in the midline represent a series of manifestations ranging from midline whitening to supraumbilical raphe, sternal cleft and sternal defects [67, 71]. One-stage surgical repair is effective for patients with PHACE syndrome with sternal defects or sternal clefts [72]. The optimal time for surgical repair ranges from 7 h after birth to 9 years of age. Repair during the neonatal period is ideal when the age of patient less than 3 months old. Because the possibility of initial closure is greater and the possibility of using prosthetic materials is lower [73]. Ventral or midline abnormalities can also occur in the chin, neck and other sites [35]. One study reported that rhabdomyomatous mesenchymal hamartoma (RMH) occurred in front of the neck and trunk in two patients with PHACE syndrome [4]. A relationship between RMH and PHACE syndrome may exist; however, a correlation between RMH and PHACE syndrome has not been reported.

#### Endocrine abnormalities

Studies reported that patients with PHACE syndrome present with endocrine abnormalities. Endocrine diseases in PHACE syndrome include hypothyroidism, pituitary hypofunction, growth hormone deficiency, thyroid hypoplasia, hypogonadism, and central adrenal insufficiency, among which thyroid dysfunction due to growth hormone deficiency and pituitary hypofunction are common abnormalities [5, 35, 44, 74, 75]. Braun et al. [41] reported that 18.3% (19/104) of patients with PHACE syndrome suffered from endocrine abnormalities. An analysis of 20 patients with endocrine abnormalities revealed that 55% (11/20) of the patients had hypothalamus‒pituitary dysfunction and that 50% (10/20) had hypothyroidism caused by thyroid dysplasia [76]. Another study reviewed the brain magnetic resonance images (MRIs) of 55 patients with PHACE syndrome and reported that 18% (10/55) of these patients had pituitary abnormalities [50]. Pituitary hypofunction is a secondary diagnostic criterion of PHACE syndrome, and it is also a rare disease feature. At present, only 5 cases have been reported [77–81]. Endocrine abnormalities are easy to ignore in PHACE syndrome patients, especially in the neonatal period [35]. When patients with PHACE syndrome have stunted growth, mental retardation, and delayed puberty, doctors should consider whether endocrine diseases are present. Therefore, clinicians need to fully understand the manifestations of endocrine abnormalities, which is very important for the early identification of endocrine abnormalities and subsequent treatment of patients with PHACE syndrome.

#### Dental issues

Some patients with PHACE syndrome have dental problems, such as hypoplasia of the enamel, abnormal roots, tooth decay, toothache, tooth loss, and delayed eruption of teeth [42, 82, 83]. One study reported that 29.8% and 15.4% of patients with PHACE syndrome had enamel defects and root problems, respectively [41]. Oral IH in PHACE syndrome may be related to hypoplasia of the enamel and abnormal roots [84]. Chiu et al. [85] reported that 45% of patients with oral IH had hypoplasia of the enamel, but Lamotte et al. [65] did not observe this phenomenon. The causes of these dental problems in PHACE syndrome remain unclear. Enamel problems increase the risk of dental caries. It is suggested that such patients should be referred to a specialist for evaluation before 1 year of age [85]. The root loss of permanent molars leads to severe consequences. Children with PHACE syndrome should undergo X-ray examination of their teeth to assess the roots of the teeth [82].

#### Extracutaneous IHs

Extracutaneous IHs are not uncommon in PHACE syndrome, which can occur in the airway, liver, gastrointestinal tract, mediastinum, lung, brain, spine, and internal auditory canal (IAC) [35, 86, 87]. Among them, airway hemangiomas are the most common, whereas IAC hemangiomas are rare [88, 89]. Airway hemangiomas are asymptomatic at birth, and approximately 80–90% appear at 6 months after birth. This is consistent with the rapid proliferation of IH, which manifests mainly as progressive hoarseness, wheezing, cough, and dyspnea [90]. Airway hemangiomas are usually found in the beard area, in which the S3 segment is at high risk [72, 91, 92]. Airway hemangiomas are potentially life-threatening manifestations of PHACE syndrome, and severe cases require tracheal intubation [93]. The incidence of airway hemangiomas varies from 40 to 52% in patients with PHACE syndrome, and most of these patients are female and white [90, 91]. These data may be underestimated because asymptomatic patients rarely undergo laryngoscopy or fiberoptic bronchoscopy.

Since the widespread use of propranolol, few patients with airway hemangiomas have undergone surgical treatment. The treatment effect of propranolol is better than that of surgery in most patients, but the average treatment time is longer [91, 94, 95]. Elluru et al. reported the experiences of 27 patients with airway hemangiomas treated with propranolol and the treatment was effective in 96% of these patients [96]. However, a study reported that an airway hemangioma subsided after treatment with propranolol and that the airway hemangioma recurred many times after the drug was stopped [97]. When severe airway obstruction is suspected, propranolol treatment should be gradually increased from the lowest dose (0.5 mg/kg/d) [98].

There is no consensus on the evaluation of airway hemangiomas, especially in patients with PHACE syndrome. Winter et al. [5] and Durr et al. [90] suggested that patients with hemangiomas with respiratory symptoms or extensive involvement of the beard should be examined via laryngoscopy and fiberoptic bronchoscopy to exclude airway hemangiomas. Moreover, a multicenter study by Czechowicz et al. [91] showed that the incidence of airway involvement was high even in patients without wheezing and other symptoms. Thus, airway evaluations are an important part of the comprehensive examination of PHACE syndrome. However, a cohort study by Ramprasad et al. [99] reported that patients with PHACE syndrome did not have adverse outcomes such as airway obstruction without airway symptoms. These findings indicate that patients with PHACE syndrome without respiratory symptoms are less likely to develop airway hemangiomas and may not need an endoscopic examination. At present, there are no guidelines to indicate whether all patients who have been evaluated for PHACE syndrome should be examined by laryngoscopy or fiberoptic bronchoscopy.

### Complications associated with PHACE syndrome

#### Headache

Headache is one of the most common complications of PHACE syndrome and often occurs in early childhood. Studies reported that 32–89% of patients with PHACE syndrome have headaches, which seriously affect their quality of life [42, 45]. Most headaches have characteristics of a migraine, including photophobia, phonophobia, nausea, and vomiting. In 2016, a study on headaches associated with PHACE syndrome reported that 62.7% of patients experienced headache symptoms and headache symptoms were more common in females, with an average age of onset of 48.8 months [100]. In 2024, Braun et al. [41] reported that 72.1% of patients with PHACE syndrome experienced headaches and nearly half experienced severe headache symptoms, which required neurological treatment. Moreover, 38.7% of patients reported having a headache that lasted more than 4 h, while the results reported by Yu et al. [100] and Stefanko et al. [42] cited values of 39.6% and 63%, respectively.

The larger the IH area of patients with PHACE syndrome is, the greater the incidence of headaches. Headaches associated with PHACE syndrome may be related to less obvious potential vascular abnormalities [41]. Currently, the etiology of headaches is unclear, and the high incidence of arterial abnormalities, cardiovascular abnormalities (including patent foramen ovale), and structural brain abnormalities (including circle of Willis variations) may increase the occurrence of headaches [6, 59, 100]. Therefore, further exploration of the pathogenesis of headaches in PHACE syndrome and their relationships with arterial abnormalities, structural brain abnormalities, and cardiovascular abnormalities is necessary.

#### Stroke

Patients with PHACE syndrome are usually at risk of progressive arterial diseases and arterial ischemic stroke (AIS), especially abnormal large blood vessels in the brain and neck [101]. Siegel et al. [49] found that AIS occurred in 15 of 22 patients with PHACE syndrome and that the age range of AIS was from 3 months to 5 years. Cerebral artery hypoplasia, hypoplasia, cerebral artery occlusion, coarctation of the aorta, severe tortuosity of blood vessels, abnormalities in multiple blood vessels, and circle of Willis are risk factors for AIS in PHACE syndrome [30, 49, 102].

Heyer et al. [64] reported that approximately 30% of patients with PHACE syndrome developed progressive arterial diseases, and some patients developed moyamoya disease and needed surgical intervention [103]. Moyamoya syndrome is a chronic progressive stenosis and occlusion disease that affects the cerebral artery and carotid artery. The etiology of moyamoya syndrome is unclear. Some investigators have suggested that moyamoya syndrome may be related to heredity and immune inflammation [104]. RNF213 variants might play a role in moyamoya disease in PHACE syndrome [3, 40, 105]. However, Hadisurya et al. [106] did not find that patients with PHACE syndrome or moyamoya disease had RNF213 variants. Studies reported that patients with PHACE syndrome can develop moyamoya disease, progressive arterial stenosis, or arterial occlusion; however, those patients do not develop AIS [45, 58, 65, 66]. The reasons may be associated with unobstructed blood vessels and adequate collateral circulation. Aspirin (3–5 mg/kg/d) is recommended for the primary prevention of stroke in high-risk PHACE syndrome [44]. At present, little is known about progressive vascular disease in PHACE syndrome. Patients at high risk of AIS need to undergo 6-branch cerebral angiography and a hemodynamic evaluation to determine whether AIS can be prevented by cerebral revascularization [107, 108].

#### Hearing loss

Hearing loss is an important manifestation of PHACE syndrome, which occurs in 17.3–42% of patients, and some patients even experience hearing loss until adulthood [41, 42, 109]. Hearing loss can be divided into sensorineural hearing loss, conductive hearing loss, and mixed hearing loss. Sensorineural hearing loss is one of the most common types of hearing loss [109]. PHACE syndrome with hearing loss need hearing aids. Moreover, most patients have IAC hemangiomas or brain abnormalities on imaging, and some patients have hearing loss due to multiple ear infections [66, 109]. Almost all hearing loss can be explained; however, otoscopy, brain magnetic resonance imaging (MRI), and other methods cannot explain the hearing loss of PHACE syndrome [65, 109]. The hearing loss associated with PHACE syndrome is usually located on the same side as that associated with cutaneous hemangiomas. The greater the range of cutaneous hemangiomas is, the greater the possibility of hearing loss [110]. Hearing loss in PHACE syndrome does not improve even with oral propranolol [109]. However, Bangiyev et al. [111] reported that sensorineural hearing loss in a patient with PHACE syndrome was reversed after oral propranolol.

At present, newborn hearing screening is widely performed. The normal hearing of patients with PHACE syndrome at birth is not a guarantee that these children will have normal hearing in the future. A comprehensive hearing evaluation should be considered for PHACE syndrome with cutaneous hemangiomas involving the ear or a large area or hearing-related imaging abnormalities. Early detection and treatment can reduce hearing loss [112].

#### Neurodevelopment impairment

Some patients have different degrees of neurodevelopmental abnormalities after one year of age, including speech difficulties, balance disorders, swallowing difficulties, hearing difficulties, learning difficulties, bradykinesia, inattention, muscle weakness, and hypotonia [42, 113]. Neurodevelopmental abnormalities associated with PHACE syndrome seem to be related to the S1 segment and abnormal brain structure [65]. Tangtiphaiboontana et al. [114] evaluated the nervous system of 29 patients older than 1 year of age with PHACE syndrome and reported that 69% had neurodevelopmental impairments, among which speech difficulties and gross motor retardation were the most common. In another study of 25 patients with PHACE syndrome, neurocognitive testing revealed that scores in most areas were comparable to those of normal children, with the exception of lower verbal scores [115]. Moreover, up to 50% of patients with PHACE syndrome experienced feeding difficulties [66]. There are limited reports of neurodevelopmental abnormalities in older children and adults with PHACE syndrome. A follow-up study of patients with PHACE syndrome over 10 years of age found that a significant proportion experienced neurodevelopmental impairments, including speech difficulties (34.6%), balance disorders (26.9%), inattention (18.3%), dysphagia (10.6%), reading disorders (9.6%), and tic disorders (5.8%) [41]. A study revealed that 67% of patients had at least one neurodevelopmental impairment [42]. Neurodevelopmental impairments significantly impact patients’ lives.To ensure early detection and intervention, parents and clinicians should prioritize the abnormal neurodevelopment in patients with PHACE syndrome. Consequently, all patients require regular clinical follow-ups and neurological assessments.

#### Psychosocial impact

Numerous studies have addressed the psychosocial impact and quality of life in patients with IH [116–120], but limited research has focused on these aspects in PHACE syndrome. In a recent study of 104 patients with PHACE syndrome, 20.2% experienced depression, 29.8% had anxiety, 32.7% reported psychological distress without medical treatment, and 45.1% exhibited learning disabilities [41]. In a study of adult patients with PHACE syndrome, 28% reported a history of depression or anxiety, and 44% exhibited learning disabilities [42]. Most patients with PHACE syndrome were satisfied with their appearance after treatment. However, the scores for the social communication ability and quality of life were lower than the general population’s average. In addition, 18.3% of patients experience IH problems, which moderately to severely affect their self-confidence, particularly in infants with IH ulcerations or those undergoing surgical treatment [41]. Parents and doctors should pay more attention to the psychosocial impact and quality of life of these patients.When patients exhibit depression or anxiety, timely medical intervention is essential to alleviate pain and lessen the burden on their families.

### Diagnosis

The diagnostic criteria for PHACE syndrome were first jointly formulated by experts in different professional fields in 2009 [6] and then updated by expert consensus in 2016 [44].

The major criteria and minor criteria for PHACE syndrome are detailed in Table 1. PHACE syndrome is defined as follows: (1) the diameter of IH on the head and face, including the scalp, is greater than 5 cm and meets 1 major criterion or 2 minor criteria; and (2) IH that occur in the neck, upper trunk, or proximal upper extremity meet 2 major criteria. Possible PHACE syndrome is defined as follows: (1) the diameter of IH on the head and face, including the scalp, is greater than 5 cm and meets 1 minor criterion; (2) IH that occur in the neck, upper trunk, or proximal upper extremity meet 1 major criterion or 2 minor criteria; and (3) no IH but meet 2 major criteria.

**Table 1:** Major and minor diagnostic criteria for PHACE syndrome

Organ systems	Major criteria	Minor criteria
Arterial anomalies	Anomaly of major cerebral or cervical arteries Dysplasia of the large cerebral arteries Arterial stenosis or occlusion with or without moyamoya collaterals Absence or moderate-severe hypoplasia of the large cerebral and cervical arteries Aberrant origin or course of the large cerebral or cervical arteries except common arch variants such as bovine arch Persistent carotid-vertebrobasilar anastomosis (proatlantal segmental, hypoglossal, otic, and/or trigeminal arteries)	Aneurysm of any of the cerebral arteries
Structural brain	Posterior fossa brain anomalies Dandy–Walker complex Other hypoplasia/dysplasia of the mid and/or hindbrain	Midline brain anomalies Malformation of cortical development
Cardiovascular	Aortic arch anomalies Coarctation of the aorta Dysplasia Aneurysm Aberrant origin of the subclavian artery with or without a vascular ring	Ventricular septal defect Right aortic arch/double aortic arch Systemic venous anomalies
Ocular	Posterior segment abnormalities Persistent hyperplastic primary vitreous Persistent fetal vasculature Retinal vascular anomalies Morning glory disc anomaly Optic nerve hypoplasia Peripapillary staphyloma	Anterior segment abnormalities Microphthalmia Sclerocornea Coloboma Cataracts
Ventral/midline	Anomaly of the midline chest and abdomen Sternal defect Sternal pit Sternal cleft Supraumbilical raphe	Ectopic thyroid hypopituitarism Midline sternal papule/hamartoma

Note: Different lesions occurring in the same organ or system, regardless of the number of lesions occurring, were counted as meeting one of the diagnostic criteria

Source: Adapted from Garzon et al.,2016 [44].

In addition, other abnormalities affecting organ systems can also be observed in PHACE syndrome. However, these abnormalities are not utilized as diagnostic criteria for PHACE syndrome (Table 2). Diagnosis relies primarily on clinical manifestations alongside imaging techniques such as MRA/MRI, echocardiography, and ophthalmologic assessments.

**Table 2:** Abnormalities outside the diagnostic criteria for PHACE syndrome

Organ systems	Abnormal manifestation
Cardiovascular	Patent ductus arteriosus, Pulmonary artery stenosis, Atrial septal defects, Ectopia cordis, Holmes’ heart
Dental	Hypoplasia of enamel, Abnormal roots, Tooth decay, Toothache, Tooth loss, Delayed eruption of teeth
Endocrine	Growth hormone deficiency, Thyroid hypoplasia, Hypogonadism, Central adrenal insufficiency, Diabetes insipidus, Hypothyroidism
Extracutaneous IH	Airway hemangioma, Liver hemangioma, Gastrointestinal tract hemangioma, Mediastinum hemangioma, Lung hemangioma, Brain hemangioma, Spine hemangioma, Internal auditory canal hemangioma, Infantile hepatic hemangioma
Eye	Ptosis, Ametropia, Horner syndrome, Amblyopia, Decreased vision, Increased intraocular pressure, Limited eye opening
Ventral/midline	Omphalocele, Rhabdomyomatous mesenchymal hamartoma, Tag-like growths

### Treatment

#### β-Blocker therapy

In 2008, Leaute-Labreze et al. [121] demonstrated that propranolol is an effective treatment for IH. Subsequently, propranolol became the first-line medication for treating problematic IH requiring systemic treatment [122–124]. What is the safety and efficacy of propranolol in treating PHACE syndrome? The evidence is inconclusive. Given that PHACE syndrome is linked to severe cerebral and carotid artery stenosis with inadequate collateral circulation, propranolol may reduce cardiac output or blood pressure, thereby heightening the risk of AIS [35, 125].

Many studies have reported that oral propranolol has good effectiveness and safety in the treatment of PHACE syndrome [27, 30, 93, 126–128]. In 2020, a study compared the safety of oral propranolol in 76 patients with PHACE syndrome and 726 patients without PHACE syndrome. No serious adverse events (such as AIS, transient ischemic attack, or cardiovascular events) associated with PHACE syndrome were observed during treatment, and no differences in nonserious adverse events were noted between patients with and without PHACE syndrome. Regardless of the risk of AIS, oral propranolol is safe and effective in patients with PHACE syndrome [58]. However, Metry et al. analyzed 32 patients with PHACE syndrome and suggested that β-blockers should be used with caution in patients at high risk of AIS [48]. Coarctation of the aorta is considered a contraindication to treatment with β-blockers, but Sharma et al. reported a patient with PHACE syndrome complicated with coarctation of the aorta. The curative effect was remarkable after oral propranolol, and no adverse events occurred [129]. The safety of propranolol in the treatment of coarctation of the aorta with PHACE syndrome needs to be investigated further. In general, patients at risk of PHACE syndrome undergo echocardiography before oral propranolol to rule out coarctation of the aorta.

Generally, it takes time to complete related tests for diagnosing PHACE syndrome. For patients with suspected PHACE syndrome, starting treatment after completing a diagnostic evaluation of PHACE syndrome may increase the risk of complications (such as ulceration and visual impairment) [12]. Therefore, a low dose of propranolol (0.5 mg/kg/d) should be used during the evaluation period to avoid large fluctuations in blood pressure [130, 131]. Patients with PHACE syndrome have longer treatment durations, and hemangiomas may recur—especially if medication is discontinued prematurely. Patients should be regularly monitored through outpatient visits, and the medication should be gradually tapered and discontinued under the supervision of a specialist [132, 133]. Patients with PHACE syndrome usually need surgery to treat other abnormalities, and oral propranolol can reduce the perioperative risk [134].

Other β-blockers (such as atenolol, nadolol, and timolol) are also widely used for the treatment of IH, and compared with propranolol, atenolol may be more effective and safer [135–137]. Theoretically, atenolol, nadolol, and timolol exert the same effects in the treatment of PHACE syndrome, but no large-scale case studies have been conducted to test that hypothesis. Only one study reported that oral atenolol was effective in the treatment of PHACE syndrome [138]. However, Pandhi et al. [139] reported that it was not safe to use timolol locally in patients with PHACE syndrome because systemic absorption might occur [140].

### Other therapies

In addition to first-line treatment with propranolol, other treatments include prednisolone, mTOR receptor inhibitors, vincristine, and lasers [35, 141]. Before propranolol was used for IH, more patients with PHACE syndrome were treated with oral prednisolone [48]. When propranolol alone or prednisolone alone is ineffective in the treatment of PHACE syndrome, combination therapy with low-dose propranolol and low-dose prednisolone may also be safe and effective [142]. Some patients with PHACE syndrome will have residual IH after oral prednisolone and/or propranolol. Laser therapy is an effective method for treating these residual IH [41, 143]. Sirolimus is an mTOR receptor inhibitor that effectively treats complex vascular malformations [144]. Kaylani et al. [145] reported a patient with refractory PHACE syndrome. Despite treatment with propranolol, prednisolone, and vincristine, the disease remained uncontrolled. Sirolimus was subsequently administered, yielding significant results. Itraconazole, an antifungal agent, inhibits angiogenesis and proliferation of infantile hemangiomas, induces apoptosis, and promotes regression [146, 147]. In 2022, a small sample IH comparative trial revealed that oral itraconazole was more effective than propranolol (44% vs. 22%) [148]. In 2024, Ahuja et al. [149] reported a 9-month-old female patient with PHACE syndrome who had contraindications for oral propranolol. Oral itraconazole (5 mg/kg/day) was administered, resulting in significant improvement and no adverse effects, such as liver damage. While these findings suggest itraconazole as a potential alternative treatment, its safety in pediatric populations remains inadequately validated, highlighting the need for future large-scale clinical and basic research to further establish its efficacy and safety.

PHACE syndrome involves various systemic abnormalities. Although propranolol and other pharmacological agents effectively treat IH, they do not address the underlying issues associated with arterial anomalies, structural brain malformations, cardiovascular anomalies, eye anomalies, and ventral/midline anomalies. Management options for these conditions are limited to observation or surgery.Currently, no alternative pharmacological treatments are available for PHACE syndrome. Comprehensive management is essential to achieve optimal therapeutic outcomes while minimizing risks. Future efforts should focus on identifying the pathogenic gene associated with PHACE syndrome and developing targeted pharmacological therapies for related gene mutations.

## Conclusions and future directions

PHACE syndrome is common in patients with segmental IH in the head and face. The prevalence of PHACE syndrome is higher in Europe and North America compared to Asia and Africa, likely influenced by ethnicity, geographic region, race, and genetic factors. PHACE syndrome usually present with multiple system abnormalities, especially arterial abnormalities and structural brain abnormalities. The quality of life in some patients is significantly impacted during both childhood and adulthood. Therefore, special attention should be given to patients with segmental IH in the head and face. Timely screening and evaluation for PHACE syndrome are essential for early diagnosis and treatment. Future research should concentrate on the pathogenesis, incidence, and risk factors of PHACE syndrome, as well as its treatment and long-term outcomes. Additional clinical studies are essential to provide more robust evidence.

## Data Availability

The data pertinent to this study are accessible through the corresponding author.

## Abbreviations

AIS: Arterial ischemic stroke
CNV: Copy number variant
HIF-1ɑ: Hypoxia inducible factor-1ɑ
IAC: Internal auditory canal
IH: Infantile hemangioma
MRA: Magnetic resonance angiography
MRI: Magnetic resonance imaging
mTOR: Mammalian target of rapamycin
PHACE: Posterior fossa anomalies, hemangioma, arterial lesions, cardiac abnormalities/coarctation of the aorta, eye anomalies
RMH: Rhabdomyomatous mesenchymal hamartoma
VEGF: Vascular endothelial growth factor
